# Characterization of Flavonoids and Phenolic Acids in *Myrcia bella* Cambess. Using FIA-ESI-IT-MS^n^ and HPLC-PAD-ESI-IT-MS Combined with NMR

**DOI:** 10.3390/molecules18078402

**Published:** 2013-07-16

**Authors:** Luiz L. Saldanha, Wagner Vilegas, Anne L. Dokkedal

**Affiliations:** 1 Botany Department, Institute of Biosciences, Univ. Estadual Paulista (UNESP), CEP 18618-970, Botucatu, Sao Paulo, Brazil; 2 Experimental Campus of the Paulista Coast, Univ. Estadual Paulista (UNESP), CEP 11330-900, Sao Vicente, Sao Paulo, Brazil; 3 Biological Science Department, Science Faculty, Univ. Estadual Paulista (UNESP), CEP 17033-360, Bauru, Sao Paulo, Brazil

**Keywords:** Brazilian savanna, flavonoid-*O*-glycosides, medicinal plants, Myrtaceae

## Abstract

The leaves of *Myrcia* DC. ex Guill species are used in traditional medicine and are also exploited commercially as herbal drugs for the treatment of diabetes mellitus. The present work aimed to assess the qualitative and quantitative profiles of *M. bella* hydroalcoholic extract, due to these uses, since the existing legislation in Brazil determines that a standard method must be developed in order to be used for quality control of raw plant materials. The current study identified eleven known flavonoid-*O*-glycosides and six acylated flavonoid derivatives of myricetin and quercetin, together with two kaempferol glycosides and phenolic acids such as caffeic acid, ethil galate, gallic acid and quinic acid. In total, 24 constituents were characterized, by means of extensive preparative chromatographic analyses, along with MS and NMR techniques. An HPLC-PAD-ESI-IT-MS and FIA-ESI-IT-MS^n^ method were developed for rapid identification of acylated flavonoids, flavonoid-*O*-glycosides derivatives of myricetin and quercetin and phenolic acids in the hydroalcoholic *M. bella* leaves extract. The FIA-ESI-IT-MS techinique is a powerful tool for direct and rapid identification of the constituents after isolation and NMR characterization. Thus, it could be used as an initial method for identification of authentic samples concerning quality control of *Myrcia* spp extracts.

## 1. Introduction

Nowadays, the main concerns about natural medicinal products are effectiveness, safety and the quality of the herbal drugs [1,2]. Consequently, it is essential to identify and measure all the bioactive constituents of medicinal plants in order to ensure the biological research reliability and repeatability as well as to ensure enhancing the quality control over the pharmacological benefits and/or hazardous. HPLC–MS plays a prominent role as an analytical tool for detecting and identifying pharmacologically active metabolites and/or reactive metabolites [3,4]. When compared to other detection methods, MS not just allows determining natural compounds chemical structure with known and unknown structures, but also offers excellent sensitivity to low amount of samples within relatively short analysis time as well as plays an important role in screening flavonoids and other phenolics [5,6,7,8].

The Brazilian savanna, called Cerrado, is included in the list of global hotspots for conservation due to its high concentrations of endemic species that had suffered heavy habitat losses [9]. Despite its relevance, the Brazilian savanna has been continuously destroyed in order to create pastures and crop fields. Nowadays, in the state of Sao Paulo, southeastern of Brazil, remnants are very reduced and fragmented [10]. The Myrtaceae is an important family in the Brazilian savanna, with more than 1,000 species countrywide and *Myrcia,* with 350 species, is one of the richest genus [11]. *Myrcia bella* is a common and important species in many savanna fragments, distributed in the state of Sao Paulo [12,13]. Indigenous and traditional communities in Brazil have used some species of this genus as astringent, against diabetes, diarrhea, diuretic, to stop bleeding, against hypertension and ulcers [14,15]. Most phytochemical studies on the *Myrcia* species are related to essential oils [16,17,18,19,20]; however Yoshikawa *et al.* [21] and Matsuda *et al.* [22] described the features of flavanone glucosides, acylated flavanone glucosides, acetophenones, flavonols and gallic acid in the leaves of *Myrcia multiflora* (Lam.) DC.

The present work aimed to assess the qualitative and quantitative profiles of *M. bella* hydroalcoholic extract, due to the potential use of the *Myrcia* species in tradittional medicine and also due to its comercial exploration as an herbal drug to be used in the treatment of diabetes mellitus [15,23,24] once the existing legislation in Brazil determines that a standard method must be developed in order to be used for quality controlling raw plant materials [25].

## 2. Results and Discussion

### 2.1. Identification of Constituents by Combination of NMR and FIA-ESI-IT-MS/MS^n^

**Figure 1:**
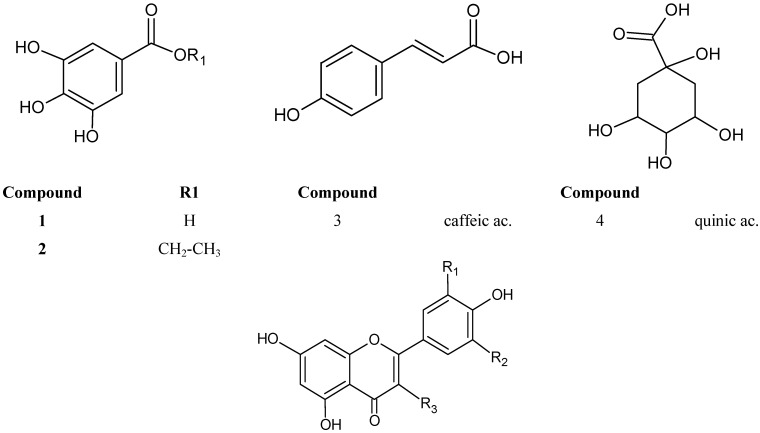
Structures of the constituents identified in the 70% EtOH leaves extract of *M. bella*.

**Figure 2:**
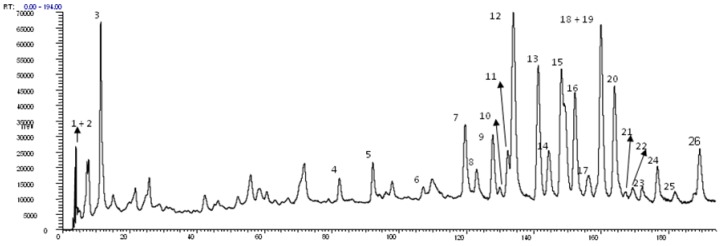
HPLC-PAD analytical chromatogram of 70% EtOH leaves extract of *Myrcia bella* with identified peaks. Experimental conditions: eluents **A** (MeOH + 0.1% form. ac.) and B (H_2_O + 0.1% Form. ac.). Gradient system: 10-45% de **A** em **B** em 200 min. Column: Phenomenex^®^ Luna C_18_ (250 × 4.6 mm i.d., 5 μm). Flow rate: 0.8 mL·min^−1^, λ = 254 nm. Injected volume: 20 μL.

In total, 24 constituents (Figure 1) were identified in the 70% EtOH extract. Eighteen of them were isolated and characterized by UV, MS and NMR spectral data and six were tentatively identified considering retention time values, co-chromatography with authentic samples, UV and MS spectral data. In Figure 2, the HPLC-PAD chromatogram of the *M. bella* 70% EtOH leaves extract is presented. Data concerning identification of the peaks are shown in Table 1, where the retention time, UV–vis absorptions and electrospray ionization mass spectrometry in negative ion mode of all the compounds detected are reported. Once this is the first experiment on chemical characterization of constituents in *M. bella*, it was necessary to completely characterize them; therefore purification and consecutive identification by 1D and 2D NMR spectroscopy was carried out.

**Table 1:** HPLC-PAD-ESI-MS data (UV-vis spectra and detected ions) and FIA-ESI-IT-MS/MS^n^ (product íons) of compounds detected in EtOH 70% leaves extract of *Myrcia bella*.

Peak (compound)	Rt (min)	UV-vis (λ_máx._)	LC-MS ions	ESI-IT-MS/MS^n^ ions	Identification	Mode of identification
1(**3**)	3.7	--	179	--	caffeic ac.	UV/MS + std
2(**4**)	4.1	--	191	191, 173, 127, 85	quinic ac.	UV/MS+ std
3	11.3	212, 278	633	--	n.i.	--
4	82.3	212, 272	635	--	n.i.	--
5	92.1	--	469	--	n.i.	--
6(**10**)	106.6	266, 356	631	479, 317, 271, 179, 151	myricetin-*O*-(*O*-galloyl)-hexoside	UV/MS + NMR
7(**7**)	119.5	266, 350	479	316, 271	myricetin-3-*O*-β-D-galactopyranoside	UV/MS + NMR
8(**9**)	123.4	266, 362	449	316, 271, 179	myricetin-3-*O*-α-arabinopyranoside	UV/MS + NMR
9(**23**)	127.8	266, 356	615	463, 301, 271	quercetin-*O*-(*O*-galloyl)-hexoside	UV/MS tentatively
10(**18**)	129.8	260, 356	615	463, 301, 271	quercetin-3-*O*-(6’’-*O*-galloyl)-β-galactopyranoside	UV/MS + NMR
11(**8**)	132.7	260, 356	449	317, 303, 271, 231, 179	myricetin-3-*O*-α-arabinofuranoside	UV/MS + NMR
12(**11**)	133.9	260, 350	463	317, 301, 271, 179, 136	myricetin-3-*O*-α-L-rhamnopyranoside	UV/MS + NMR
13(**12**)	141.4	260, 350	463	301, 273, 179, 151	quercetin-3-*O*-β-D-galactopyranoside	UV/MS + NMR
14(**21**)	144.3	254, 362	463	301, 273, 179, 151	quercetin-O-hexoside	UV/MS tentatively
15(**16**)	148.5	260, 356	433	301, 261, 191	quercetin-3-*O*-β-D-xylopyranoside	UV/MS + NMR
16(**15**)	152.3	254, 356	433	301, 261, 191	quercetin-3-*O*-β-D-xylofuranoside	UV/MS + NMR
17(**22**)	155.9	266, 352	601	449	myricetin-*O*-(*O*-galloyl)-pentoside	UV/MS tentatively
18	159.7	--	467	--	n.i.	--
19(**17**)	160.0	266, 356	433	301, 191	quercetin-*O*-α-L-arabinofuranoside	UV/MS + NMR
20(**14**)	164.0	254, 350	447	301, 271, 255, 179	quercetin-3-*O*-α-L-rhamnopyranoside	UV/MS + NMR
21	163.8	--	483	--	n.i.	--
22	169.2	--	477	--	n.i.	--
23(**24**)	171,0	266, 356	615	463, 317	myricetin-*O*-(*O*-galloyl)-deoxyhexoside	UV/MS tentatively
24(**20**)	176.8	260, 356	585	433, 301, 179, 151	quercetin- *O*-(*O*-galloyl)-pentoside	UV/MS + NMR
25	181,0	--	631	--	n.i.	--
26(**19**)	189.4	254, 374	301	137	quercetin	UV/MS + NMR

* n.i.: not identified; std: standard.

Diagnostics mass fragments obtained by FIA-ESI-IT-MS in the negative mode at 285, 301 and 317 characterized aglycones as kaempferol, quercetin and myricetin, respectively. The precursor ion at 169 mu characterized gallic acid. The neutral losses of 132, 162, 146 and 152 mass units allowed the identification of pentosides (xylose or arabinose), hexosides (glucose or galactose), deoxyhexoside (rhamnose) and gallic acid respectively. The values of *m/z* lower than the aglycone (*i.e.*, *m/z* < 317) like *m/z* 179 (^1,2^A^−^), 151 (^1,3^A^−^) and 137 (^1,2^B^−^) are typical of retro Dies-Alder (RDA) reactions of flavon-3-ols having a dihydroxylated A ring and *m/z* 137 is a typical fragment of the trihydroxylated B ring [26].

The HPLC-PAD analysis of the chromatogram peaks with bands at 210–278 nm (0–100 min) were related to the presence of phenolic acids and bands at 240–285 nm and 350–380 nm (100–180 min) were related to flavonoids. 

The signals in *m/z* 169 e 197 were diagnostic for compounds **1** and **2** respectively. The ^1^H-NMR spectra were consistent with the data obtained by MS. ^1^H-NMR experiments of compounds **11** and **14** presented diagnostic signals at δ 0.83 and ^13^C-NMR signal at δ 17.51 for rhamnoses [27,28]. The anomeric proton signal indicated the configuration α-l-rhamnopyranoside and HMBC experiments of compound **14** showed the anomeric proton correlation at δ 5.25 with C3 at δ 134.1, confirming the position of sugar linkage.

^1^H-NMR data of compounds **15**, **16** and **17** were in agreement with typical quercetin chemical shifts. The anomeric proton of compound **17** at δ 5.18 (*J* = 3.5 Hz) indicated the presence of α-l-arabinofuranoside. The anomeric proton at δ 5.32 (*J* = 7.3 Hz) indicated the presence of β-d-xylopyranoside in compound **16** and at δ 5.27 (*J* = 1.5 Hz) the presence of β-d-xylofuranoside in compound **15**. ^13^C-NMR data of sugar unit were in agreement to the literature [29,30,31,32].

The loss of 152 Da in MS spectra of compound **18** suggested the presence of a galloyl unit. ^13^C-NMR signals were in agreement to MS data and confirm the presence of galloyl unit [29].

**Figure 3:**
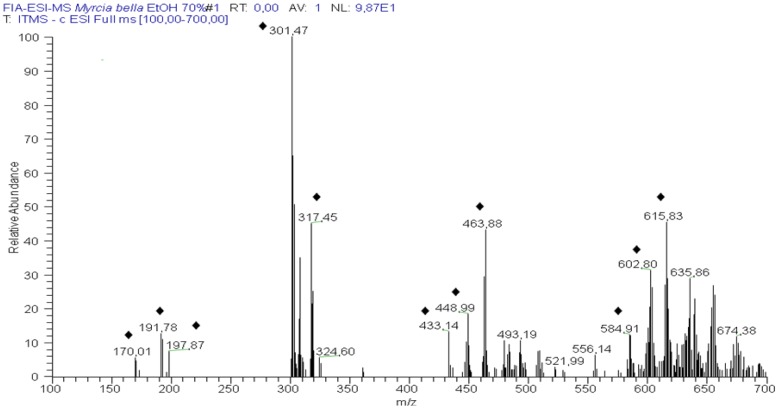
Typical direct flow injection analysis FIA-ESI-IT-MS fingerprint spectra obtained in negative ion mode of the 70% EtOH from the leaves of *M. bella*. (♦) Representative constituents fragmented. For conditions, see Material and Methods part.

In addition to the experiments which led to the isolation and identification of compounds, an analysis was also carried out to confirm the chemical composition of the 70% EtOH extract employing the combination of multi-stage analysis of selected ions by ESI-IT-MS/MS^n^ and HPLC-PAD-ESI-MS. The main objective of this analysis was to evaluate the ability of this technique to produce spectral information on the chemical constitution sample analyses quickly and directly, without the need for pretreatment steps and/or chromatographic separations. Six constituents (compounds **3**–**4** and **21**–**24**) were tentatively identified. Within the exception of the isolated compounds **1**, **2**, **5**, **6** and **12**, all other compounds were simultaneously identified by HPLC-PAD-ESI-MS. The typical precursor ions spectrum in negative ion mode of 70% EtOH leaves extract of *M. bella* is presented in Figure 3.

The precursor ion at *m/z* 197 [M – H]^−^ was consistent with the presence of compound **2**. The fragmentation of the precursor ion at *m/z* 169 [M – H]^−^ produced the ion at *m/z* 125 [M – 44 – H]^−^, due the loss of 44 Da (COO^−^), thus confirming the presence of compound **1** [33]. The precursor ion at *m/z* 191 [M – H]^−^ produced fragment ions at *m/z* 172, *m/z* 127 and *m/z* 85. This pattern of fragmentation led to the identification of quinnic acid (**4**). Precursor ions at *m/z* 301 [M – H]^−^ and *m/z* 317 [M – H]^−^ in the precursor ion spectrum were in agreement to the presence of compounds **21** and **14**.

**Figure 4:**
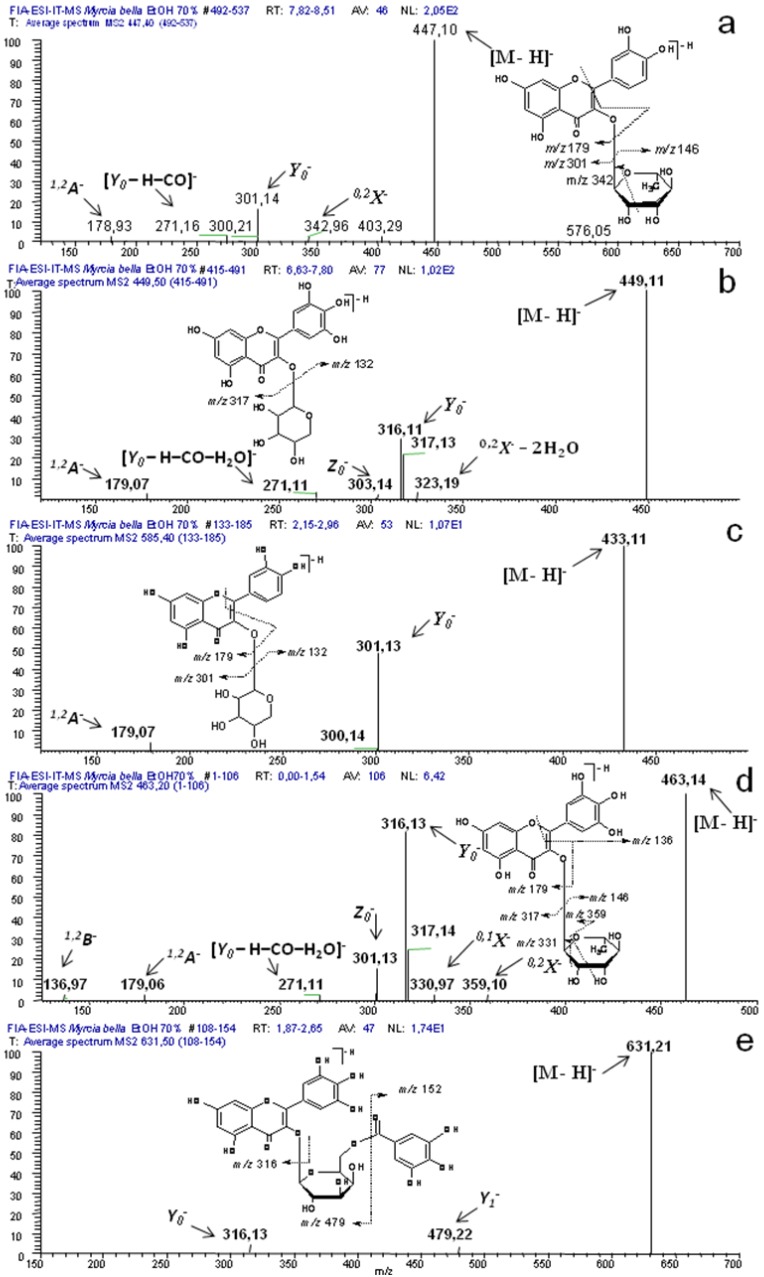
Second-generation product ion spectra obtained for the main precursor ions produced in the FIA-ESI-MS experiment and the proposed fragmentation. For conditions, see the Experimental part.

Figure 4 presents the MS^n^ fragmentation of representative compounds from *M. bella*. The nomenclature for the flavonoids was that of Ma *et al.* [26] and product ions from glycoconjugates were denoted according to the nomenclature introduced by Domon and Costello [27].

Second-generation product ion spectra of precursor ion at *m/z* 447 [M – H]^−^ (Figure 4a), produced the product ion *Y_0_*^−^ at *m/z* 301 [M – 146 – H]^−^ and the diagnostic product ion ^0,2^*X*^−^ due to the loss of 104 Da resulting from the cleavage of the sugar unit, typical of deoxyhexoses [28] as well as [*Y_0_* – H – CO]^−^ at *m/z* 271 typical of flavon-3-*O*-monoglycoside [34] and at *m/z* 179 from the RDA of ring A. Such fragments confirmed the presence of compound **14**.

The second-generation of the precursor ion at *m/z* 449 [M – H]^−^ (Figure 4b) produced the product ion *Y_0_*^−^ at *m/z* 316 [M – 132 – 2H]^−^ and ^0,2^*X*^−^ – 2H_2_O at *m/z* 323 after loss of 126 Da, typical of pentoses, as well as the product ions ^1,2^*A*^−^ at *m/z* 179, [*Y_0_* – H – CO – H_2_O]^−^ at *m/z* 271 and *Z_0_*^−^ at *m/z* 303, resulting from the RDA of ring A, thus confirming the presence of compounds **8** and **9**.

The second-generation product ion spectra of precursor ion at *m/z* 433 [M – H]^−^ (Figure 4c) produced the ion product *Y_0_*^−^ at *m/z* 301 [M – 132 – H]^−^, due the loss of 132 Da and ^1,2^*A*^−^ at *m/z* 179 resulting from the RDA of ring A. Such fragmentation pattern is in agreement to componds **17**, **18** and **19**.

The second-generation of the precursor íon at *m/z* 479 [M – H]^−^ produced the product ion *Y*_0_^−^ at *m/z* 316 [M – 162 – 2H]^−^, after loss of 162 Da. The product ion [*Y_0_* – H – CO – H_2_O]^−^ at *m/z* 271 is typical of 3-*O*-monoglycosides [34]. This fragmentation pattern confirms the presence of compound **9**.

The second-generation of the precursor ion at *m/z* 463 [M – H]^−^ (Figure 4d) produced the product ion *Y*_0_^−^ at *m/z* 316 [M – 146 – 2H]^−^ as well as the products ion ^0,2^*X*^−^ at *m/z* 359 [M – 104 – H]^−^ and ^0,1^*X*^−^ at *m/z* 331 [M – 132 – H]^−^ resulting from the cleavage of the sugar unit, typical of deoxyhexoses [34]. Other fragments such as *Y*_0_^−^, *Z*_0_^−^ at *m/z* 301 [M – 146 – 18 – H]^−^, [*Y_0_* – H – CO – H_2_O]^−^ at *m/z* 271, ^1,2^A^−^ at *m/z* 179 and ^1,3^B^−^ at *m/z* 136 were in agreement to the presence of compound **11**.

Fragmentation of the precursor ion at *m/z* 631 [M – H]^−^ (Figure 4e) produced the ion product *Y*_1_^−^ at *m/z* 479 [M – 152 – H]^−^. Fragmentation of *Y*_1_^−^ produced the product ion *Y*_0_^−^ at *m/z* 316 [M – 152 – 162 – 2H]^−^. Such fragmentation pattern confirms the presence of compound **10**.

HPLC-PAD-ESI-MS revealed the presence of two peaks more with the precursor ion at *m/z* 463 at later retention time. The second-generation of the precursor ion at *m/z* 463 [M – H]^−^ produced the product ion *Y*_0_^−^ at *m/z* 301 [M – 162 – H], suggesting the presence of quercetin derivatives. Co-chromatography with the isolated compounds confirms the presence of compound **13** at 141.4 min and allowed the identification of quercetin-*O*-hexoside (**21**) at 144.3 min.

The third-generation of the precursor íon at *m/z* 585 [M – H]^−^ produced products ions *Y*_1_^−^ at *m/z* 433 [M – 152 – H]^−^ and *Y*_0_^−^ at *m/z* 301 [M – 132 – 152 – H]^−^. The fragment ^1,2^*A*^−^ at *m/z* 179 resulted from the RDA elimination of the ring B typically of quercetin. Such results were in agreement with the presence of compound **20**.

Myricetin-*O*-(*O*-galloyl)-pentoside (**22**) was assigned to a peak **17** with a retention time of 155.9 min and showed the precursor ion at *m/z* 601 [M – H]^−^. The second-generation spectra shows the product ion*Y*_0_^−^ at *m/z* 449 [M – 152 – H]^−^ after the loss of 152 Da attributed to a galloyl-glycoside moiety and UV maximum was 266, 355 nm.

HPLC-PAD-ESI-MS analysis revealed the presence of three peaks (**9**, **10** and **23**) with different retention times with precursor ion at *m/z* 615. The second-generation product ion spectra of the precursor ion at *m/z* 615 [M – H]^−^ produced the products ions at *m/z* 463 [M – 152 – H]^−^, *m/z* 301 [M – 152– 162 – H]^−^ and *m/z* 317 [M – 152– 146 – H]^−^. Such fragmentation pattern confirmed the presence of compound **18** and co-chromatography in combination to spectral data indicated the presence of quercetin-*O*-(*O*-galloyl)-hexoside (**23**) at earlier retention time of 127.8 min and myricetin-*O*-(*O*-galloyl)-deoxyhexoside (**24**) at later retention time of 171.0 min.

Finally, the multi-stage analysis of selected ions by FIA-ESI-IT-MS/MS^n^ provided rich information of the compounds of the 70% EtOH extract providing the identification of minor compounds not isolated without purification or pre-treatment.

### 2.2. Validation Data

#### 2.2.1. Linearity, Repeatability of the Standards, LOD, LOQ and Precision

All compounds showed good linearity. The following r^2^ values were obtained: gallic acid r^2^ = 0.9992 (regression curve: y = 75639x −2.2237) and quercetin r^2^ = 0.9996 (regression curve: y = 87488x −3.31288). LOD for gallic acid was 2.27 µg·mL^−1^ and LOQ was 6.8 µg·mL^−1^ (10 μL of injection). For quercetin, LOD was calculated as 1.57 µg·mL^−1^ and LOQ was 4.75 µg·mL^−1^.

The repeatability, based on three samples with known concentration, was analyzed by HPLC and the relative standard deviation (% RSD) of the standards was calculated. RSD values ranged between 0.32 and 3%. The overall intraday time variations of the standards were less than 0.32–2.40% for gallic acid and 0.39–3.0% for quercetin and interday time variations were less than 0.52–2.32% for gallic acid and 0.47–1.47% for quercetin. Precision data are displayed in Table 2.

**Table 2:** Precision data of the two analytes, expressed as RSD (%).

Analytes	Concentration (μg·mL^−1^)	Precision			
		Intra-day (mean ± SD)	RSD %	Inter-day (mean ± SD)	RSD %
Gallic ac.	50	41.66 ± 1.0	2.40	35.93 ± 0.85	2.36
(n = 3)	100	77.53 ± 0.25	0.32	74.16 ± 0.49	0.66
	200	143.2 ± 0.95	0.66	142.13 ± 0,75	0.52
Quercetin	50	45.23 ± 0.30	0.66	42.63 ± 0.63	1.47
(n = 3)	100	85.2 ± 2.6	3.0	86.43 ± 0.81	0.93
	200	163.36 ± 0.64	0.39	169.73 ± 0.8	0.47

**Table 3:** Estimative of contents of phenolic acids and flavonoids glycosides derivatives in 70% EtOH leaves extract (n = 3) of *M. bella* expressed by the use of gallic acid and quercetin linear regression data.

Compound	Concentration ± SD (µg.mL^−1^)	Standard
gallic ac.	12.13 ± 2.35	GA
n.i.	7.19 ± 0.87	GA
n.i.	8.07 ± 0.71	GA
n.i.	8.40 ± 0.57	GA
Myricetin-3-*O*-β-D-galactopyranoside	9.92 ± 0.91	Q
Quercetin-O-(6’’-*O*-galloyl)-β-galactopyranoside	5.46 ± 0.65	Q
Quercetin-3-O-β-D-galactopyranoside	21.82 ± 2.05	Q
Quercetin-*O*-hexoside	11.09 ± 1.05	Q
Quercetin-3-*O* β-D-xylopyranoside	7.01 ± 0.78	Q
Quercetin-3-*O*-β-D-xylofuranoside	14.06 ± 1.33	Q
Quercetin-3-*O*-α-L-arabinofuranoside	29.99 ± 3.37	Q
Quercetin-*O*-α-L-rhamnopyranoside	15.81 ± 1.49	Q
Quercetin	9.83 ± 1.47	Q
Phenolic acid estimative	35.80	
Flavonoids estimative	129.02	

* n.i.: compound not identified; GA = gallic acid, Q = quercetin; Contents (µg·mL^−1^) corresponding to 10 mg·mL^−1^ of 70% EtOH leaves extract sample.

### 2.3. Quantitative Analysis

In a first look at the chromatogram it became evident that the main constituents were quercetin glycosides derivatives containing one sugar unit. In order to make the most pratical method, we used two reference standards available on the market. Quercetin glycosides derivatives were expressed as quercetin, whereas phenolic acids derivatives were expressed as gallic acid. The quantitative analysis results are reported in Table 3.

## 3. Material and Methods

### 3.1. Solvents and Chemicals

The solvents used on HPLC analysis were HPLC grade; MeOH and formic acid (85% v/v) for HPLC were purchased from Merck (Sao Paulo, Brazil). Water was purified by a Milli-Q plus system from Millipore^®^. PTFE membrane filter (0.45 mm) was also purchased from Merck. All laboratory chemicals used in the current study in the isolation protocol were of reagent grade. 

### 3.2. Plant Material

Samples of *M. bella* leaves were collected in November 2010 at the Botanical Garden of Bauru (22°20'30" S e 49°00'30" W)—SP, Brazil. Voucher specimens were prepared and identified by A. L. Dokkedal and stored at the Herbarium of the UNESP—Univ Estadual Paulista “Júlio de Mesquita Filho”—UNBA (Bauru—SP, Brazil) under code number 5508.

### 3.3. Standards

The following standards were used for quantitative analysis: gallic acid and quercetin, which were purchase from Sigma-Aldrich (Sao Paulo, Brazil).

### 3.4. Isolation of the Characteristic Constituents

Fresh leaves were dried at 40 °C for 48 h. The separated powdered leaves (1.3 kg) were extracted with EtOH-H_2_O (7:3) by percolation at room temperature for 2 months [35]. The ethanolic solution was filtered and concentrated to dryness under reduced pressure at 40 °C furnishing 364 g of the hydroalcaholic extract (70% EtOH); a portion (15 g) of this extract was redissolved in MeOH-H_2_O (1:4) and partitioned with equal volumes of *n*-hexane, dichloromethane, *n*-butanol and water (Fr_1_–Fr_4_) to obtain four major fractions. HPLC-PAD analysis showed that fraction Fr_3_ was richer in flavonoid glycosides. Fraction Fr_3_ (3 g) was dissolved in 15 mL of MeOH and subjected to size exclusion chromatography using a Sephadex LH-20 column (85 × 2.5 cm; H × i.d.) with peristaltic pump and automatic collector Redifrac using MeOH as mobile phase yielding 366 fractions (10 mL each) (Mb_1_–Mb_366_). Fractions Mb_77_-Mb_366_ were combined by similarity and were subjected to purification by semipreparative HPLC-RI (Knauer^®^ 2300) using a C_18_ (250 × 10.0 mm i.d.; 5 μm) column and as mobile phase MeOH-H_2_O (4:6). Successive chromatographic steps of the above subfractions afforded 9 mg of gallic acid (**1**), 7 mg of ethyl gallate (**2**), 3 mg of kaempferol-*O*-deoxyhexoside (**5**), 2 mg of kaempferol-*O*-hexoside (**6**), 12 mg of myricetin-3-*O*-β-d-galactopyranoside (**7**), 4 mg of myricetin-3-*O*-α-arabinofuranoside (**8**), 26 mg myricetin-3-*O*-α-arabinopyranoside (**9**), 3 mg of myricetin-*O*-(*O*-galloyl)-hexoside (**10**), 16 mg of myricetin-3-*O*-α-l-rhamnopyranoside (**11**), 18 mg myricetin (**12**), 19 mg of quercetin-3-*O*-β-d-galactopyranoside (**13**), 25 mg of quercetin-3-*O*-α-l-rhamnopyranoside (**14**), 8 mg of quercetin-3-*O*-β-d-xylofuranoside (**15**), 5 mg of quercetin-3-*O*-β-d-xylopyranoside (**16**), 13 mg of quercetin-3-*O*-α-l-arabinofuranoside (**17**), 16 mg of quercetin-3-*O*-(*6’’*galloyl)-β-galacto-pyranoside (**18**), 15 mg of quercetin (**19**) and 4 mg of quercetin-*O*-(*O*-galloyl)-pentoside (**20**).

### 3.5. HPLC-PAD Analysis Instrumentation

The HPLC system consisted of a PU-2089S Plus (Jasco^®^) pump equipped with a MD-2015 Plus Photodiode Array Detector (PAD, Jasco^®^) and AS-2055 automatic injector (Jasco^®^). The column was a Luna C_18_ column (250 × 4.6 mm, i.d.) with a particle size of 5 mm (Phenomenex^®^) maintained at 35 °C, and managed by the Jasco *ChromPass* software. The eluents were: **A** (MeOH + 0.1% formic acid) and **B** (H_2_O + 0.1% formic acid.). The gradient condition was: 5–45% of **A** in **B** in 190 min. Injected volume of the samples was 20 μL solution. The UV–vis spectra were recorded between 200 and 600 nm, and the chromatographic profiles were registered at 254, 280 and 360 nm.

### 3.6. FIA-ESI-IT-MS/MS^n^ and HPLC-ESI-IT-MS Analysis Instrumentation

The chromatographic profile of the crude extract of *M. bella* was performed using Accela High Speed LC (Thermo Scientific^®^, San Jose, CA, USA), Phenomenex^®^ Luna C_18_ (250 × 4.6 mm i.d.; 5 μm) column and (Phenomenex^®^) 4 × 3 mm i.d., column guard, with PAD and coupled to an Accela (Thermo Scientific^®^) LCQ Fleet with Ion Trap (IT) 3D and ionization by eletrospray (ESI). Mobile phase was Water ultra pure (eluent **A**) and Methanol (eluent **B**), both containing 0.1% of formic acid. The ratio was: 10–45 of **A** in **B** in 190 min. Injection volume: 20.0 μL; Column temperature: 25 °C; Flow ratio: 0.8 mL·min^−1^; the chromatogram was monitored at 254 nm. The effluent from the HPLC was directed into the ESI probe. 

Using this method, we determined the most intense parent ion for each peak in the chromatogram. A second event, FIA-ESI-IT-MS in the negative ion mode, was performed using the same equipment described above, equipped with Xcalibur software. The 70% EtOH extract was dissolved in MeOH-H_2_O (8:2) and infused in the ESI source by flow injection analysis (FIA) using a syringe pump; the flow rate was 33 μL·min^−1^. The capillary voltage was −20 V, the spray voltage was 4 kV, and the tube lens offset was −55 V. The capillary temperature was 275 °C. Nitrogen was used both as drying gas at a flow rate of 60 (arbitrary units) and as nebulising gas. The nebulizer temperature was set at 280 °C, and a potential of −4 V was used on the capillary. Negative ion mass spectra were recorded in the range *m/z* 100–1,550. Two scan events were prescribed to run in the LCQ mass spectrometry. The first event was a full-scan spectrum to acquire data on the deprotonated compounds within the scan range. The second scan event was a MS/MS experiment performed using a datadependent scan on deprotonated molecule [M − H]^−^. The collision energy for MS/MS was adjusted to 10–25%.

### 3.7. Identification of Peaks and Peak Purity

Identification of all constituents was performed by HPLC-PAD and MS analysis by comparing the retention time, the UV and MS spectra of the peaks in the samples with those of authentic reference samples or isolated compounds. The purity of peaks was checked by a PAD coupled to the HPLC system, comparing the UV spectra of each peak with those of authentic references samples and/or by examination of the MS spectra.

### 3.8. Linearity Limitation of Detection, Limitation of Quantification and Precision

The optimized HPLC-PAD method was validated for the simultaneous analysis of gallic acid and quercetin in terms of linearity, limit of detection, limit of quantification, precision and accuracy. The calibration curves were obtained by the external standard method on eight levels of concentration of standard mixtures, with three injections per level. Chromatogram peak areas at 280 nm for gallic acid and at 360 nm for quercetin were plotted against the known concentrations of the standard solutions to establish the calibration equations. A linear regression equation was calculated by the least squares method. The detection limit (LOD) and limit of quantification (LOQ) were calculated from the residual standard deviation of the regression (σ) line and the slope (S) as follows: LOD = 3.3σ/S; LOQ = 10σ/S. Three different concentrations of standard mixtures (0.05; 0.1 and 0.2 mg·mL^−1^) were used for intra- and interday precision testing. The areas under curves and retention times of the three consecutive injections, performed at each concentration on three different days, were used to calculate % RSD (relative standard deviation) interday precision. Intraday precision data for peak areas and retention times were calculated from six non-consecutive injections, performed at each concentration on the same day. 

### 3.9. Quantitative Determination of Constituents

The method of external standard was applied to quantify each compound. Quantification of individual constituents was performed using a regression curve, each point in triplicate. Measurements were performed at 360 nm, which is the maximum absorbance for flavonols and 280 nm for phenolic acids.

### 3.10. NMR Analysis

The ^1^H-NMR and ^13^C-NMR 1D and ^1^H-NMR 2D-NMR ^13^C *g*-HMBC experiments were performed on a Bruker^®^ 300 MHz (7.0 T) nuclear magnetic resonance spectrometer and/or on a Varian Inova^®^ 500 MHz (11.7 T) nuclear magnetic resonance spectrometer (for sample preparation for the NMR experiments pyridine-d_5_ and dimethyl sulfoxide (DMSO-d_6_, Cambridge Isotope Laboratories, Inc., Andover, MA, USA) were used. 

## 4. Conclusions

The qualitative and quantitative profiles of the 70% EtOH extract from the leaves of *M. bella* were analyzed. The combined NMR, FIA-ESI-IT-MS/MS^n^ and HPLC-PAD-ESI-MS techniques allowed the unambiguously identification of 24 compounds. Eighteen constituents were isolated and confirmed by NMR and/or MS analysis and six were tentatively identified. Main secondary metabolites were flavonoid glycosides, mainly derivatives of quercetin and myricetin. The HPLC-PAD analysis also revealed the presence of peaks with typical UV spectra of phenolic acids derivatives in the chromatogram. The genus is reported to contain flavonol-*O*-glycosides of myricetin and quercetin as well as flavanones glucosides and acetophenone glucosides in polar extracts [20,21,22]. The FIA-ESI-IT-MS techinique is a powerful tool for direct and rapid identification of the constituents after isolation and NMR characterization. Thus, it could be used as a starting method for identification of authentic samples for the purposes of quality control of *Myrcia* spp extracts. 
